# Comparison of the Associations of Early-Life Factors on Wheezing Phenotypes in Preterm-Born Children and Term-Born Children

**DOI:** 10.1093/aje/kwy268

**Published:** 2019-01-22

**Authors:** Sarah J Kotecha, W John Watkins, John Lowe, Raquel Granell, A John Henderson, Sailesh Kotecha

**Affiliations:** 1 Department of Child Health, School of Medicine, Cardiff University, Cardiff, United Kingdom; 2 Population Health Sciences, Bristol Medical School, University of Bristol, Bristol, United Kingdom

**Keywords:** Millennium Cohort Study, prematurity, wheeze

## Abstract

Although respiratory symptoms, including wheezing, are common in preterm-born subjects, the natural history of the wheezing phenotypes and the influence of early-life factors and characteristics on phenotypes are unclear. Participants from the Millennium Cohort Study who were born between 2000 and 2002 were studied at 9 months and at 3, 5, 7, and 11 years. We used data-driven methods to define wheezing phenotypes in preterm-born children and investigated whether the association of early-life factors and characteristics with wheezing phenotypes was similar between preterm- and term-born children. A total of 1,049/1,502 (70%) preterm-born children and 12,307/17,063 (72%) term-born children had recent wheeze data for 3 or 4 time points. Recent wheeze was more common at all time points in the preterm-born group than in term-born group. Four wheezing phenotypes were defined for both groups: no/infrequent, early, persistent, and late. Early-life factors and characteristics, especially antenatal maternal smoking, atopy, and male sex, were associated with increased rates for all phenotypes in both groups, and breastfeeding was protective in both groups, except late wheeze in the preterm group. Preterm-born children had similar phenotypes to term-born children. Although early-life factors and characteristics were similarly associated with the wheezing phenotypes in both groups, the preterm-born group had higher rates of early and persistent wheeze. However, a large proportion of preterm-born children had early wheeze that resolved with time.

Due to increased respiratory symptoms and lower attainment of peak lung function in preterm-born children, there have been concerns that they might be at risk of premature development of chronic obstructive pulmonary disease (1). Although it has been established that very preterm-born children, born at <32 weeks’ gestation, have longer-term lung function deficits (2), especially if they develop bronchopulmonary dysplasia in infancy (also called chronic lung disease of prematurity (3)), it is increasingly recognized that even those born late preterm (33–36 weeks’ gestation) have lung function deficits (4, 5). Two recent longitudinal studies have reported that lung function declines with age (6, 7). Doyle et al. (6) reported the longitudinal outcomes at 8 and 18 years of life of infants born at <28 weeks’ gestation or <1,000 g in a cohort born after surfactant was introduced. Deficits in lung function increased between the 2 time points when compared with term controls. Simpson et al. (7) studied children born at ≤32 weeks’ gestation in early and mid-childhood and again reported declines in lung function between the 2 time points, compared with term controls. Respiratory symptoms are also increased, showing a gradient of increasing wheeze with increasing prematurity (8, 9). Even those born early term (37–38 weeks’ gestation) have greater respiratory symptoms than children born at 39–40 weeks’ gestation (10, 11).

In asthma, a complex heterogeneous disease, a number of wheezing phenotypes have been described based on wheezing patterns over time (12–15). It is important to accurately define wheezing phenotypes, because some, such as persistent wheeze, are associated with longer-term decrements in lung function; furthermore, different underlying mechanisms or endotypes might be associated with the different wheezing phenotypes (12, 14, 16, 17). After preterm birth, it has been assumed that long-term respiratory outcomes are the consequence of dysregulated lung growth (18, 19) and neonatal treatment, with early wheezing in infancy persisting into adulthood without those individuals ever reaching optimal lung function (1). Simpson et al. (7) studied respiratory symptoms from early to mid-childhood, and symptoms remained consistent. However, thus far, no longitudinal studies have, to our knowledge, reported on the wheezing phenotypes in preterm-born children. Wheezing phenotype studies have included preterm-born children in their cohorts but have not specifically examined them (13, 14, 20). In addition, the association of early-life factors and characteristics with wheezing phenotypes in preterm-born children is unclear. We postulated that preterm-born children differ from term-born children because they are born at an earlier stage of lung development and might have greater noxious exposures in early life (e.g., to supplemental oxygen, mechanical ventilation, and neonatal infections, among other exposures) compared with term-born children. Wheezing illnesses are heterogeneous in early childhood, and only some are associated with asthma in later life. Therefore, asthma and wheezing cannot be segregated. Although we know some of the long-term consequences of preterm birth, there might be other endotypes that are important in the development of wheezing illnesses in this population. It is important to identify these associations, because this could potentially lead to the identification of preterm-born children at risk of the different wheezing phenotypes. If any early-life factor or characteristic is modifiable, then long-term respiratory symptoms might be modifiable. Therefore, using a well-established cohort, we: 1) defined the different wheezing phenotypes in preterm-born children, comparing results with term-born children; and 2) identified and compared the association between early-life factors and characteristics and the different wheezing phenotypes in the term- and preterm-born groups separately.

## METHODS

### Millennium Cohort Study

The Millennium Cohort Study (MCS) is a cohort of 19,517 children born in the United Kingdom between 2000 and 2002, as previously described (21, 22). The data for all MCS sweeps is available to download from the UK Data Service (23). All data were collected at face-to-face interviews as described in Web Appendix 1 (available at https://academic.oup.com/aje). At 9 months of the child’s age, data were collected on pregnancy, birth and early-life factors, and characteristics and at 3, 5, 7, and 11 years of age on respiratory symptoms (including “wheeze—ever” and “recent wheeze,” defined as parental reporting of wheezing or whistling in the chest in the previous 12 months). Recruitment, ethical approval, and parental consent were obtained as described previously (24).

### Statistical analyses

Preterm- and term-born were defined as birth at <37 and ≥37 weeks’ gestation, respectively. Birth-weight *z* scores were calculated using the LMSgrowth program (25), correcting for gestation and sex. Intrauterine growth restriction (IUGR) was defined as <10th centile for birth weight, corrected for sex and gestational age (26, 27). Demographic factors and wheezing symptoms were compared between the preterm and term groups using independent sample *t* tests or χ^2^ tests.

Wheezing phenotypes were derived from respiratory symptoms reported on at least 3 occasions. Latent GOLD (Statistical Innovations, Boston, Massachusetts) was used to estimate latent class cluster models by data-driven methods, as described in Web Appendix 1. The class posterior probability was used to assign each wheezing pattern to the class of wheezing phenotype that it had the highest probability of belonging to, using the probabilities specified by the Latent GOLD analysis. Demographic factors for the different wheezing phenotypes were compared using analysis of variance or χ^2^ tests. Using cases with complete data for the early-life factors and characteristics for both the preterm group and the term group, we conducted a multinomial logistic regression with wheezing phenotype as the outcome variable, using the no/infrequent-wheeze class as the reference group. Early-life factors and characteristics that have been reported to have a direct association on preterm birth or on later wheezing were chosen (Web Figure 1) (28–31). All the parameters were included in an initial multivariable model, and only the parameters with a suggestive evidence of association based on a threshold of *P* < 0.1 were included in the final multivariable model.

Analyses were performed using Latent GOLD, version 5.1 (Statistical Innovations), and PASW 20 (SPSS Inc., Chicago, Illinois).

## RESULTS

From a total of 19,244 families, data were available from 18,552 (96.4%), 15,590 (81.0%), 15,246 (79.2%), 13,857 (72.0%), and 13,287 (69%) families at 9 months and 3, 5, 7, and 11 years, respectively. From 19,517 children in the original cohort, 18,565 (95.1%) had data on gestational age; 1,502 (8.1%) children were born preterm, of whom 1,049 (69.8%) had recent-wheeze data for at least 3 time points and thus were included in the phenotype analyses. From 17,063 term-born children, data for phenotype analyses were available for 12,307 (72.1%). Web Table 1 compares included and excluded children (results are discussed in Web Appendix 2), and Table 1 compares included preterm- and term-born children. Included preterm-born children had lower birth weight and gestational age, were less likely to be breastfed, and had fewer siblings and lower rates of formal childcare than included term-born children. However, they had higher rates of IUGR, cesarean delivery, neonatal unit admissions, hospital stays, asthma diagnosis, and antenatal maternal smoking, and the body mass index of a larger percentage of mothers of preterm-born children was outside the normal range, compared with mothers of term-born children. Socioeconomic status was similar.

**Table 1.** Demographic Characteristics of Preterm-Born and Term-Born Children, Born During 2000–2002, Who Had Wheezing Phenotype Data, Millennium Cohort Study, United Kingdom

Characteristic	Preterm Born (*n* = 1,049)			Term Born (*n* = 12,307)			*P* Value
	No.	%	Mean (SD)	No.	%	Mean (SD)	
Mean birth weight, kg^a^			2.33 (0.68)			3.43 (0.51)	0.00
Mean birth-weight *z* score			0.01 (1.20)			−0.03 (1.00)	0.18
Mean gestation, weeks^a^			34.3 (2.4)			39.8 (1.3)	0.00
24–28 weeks’ gestation	50	4.8					
24–32 weeks’ gestation	192	18.3					
Male sex	536	51.1		6,202	50.4		0.66
IUGR at birth^a,b,c^	145	13.8		1,238	10.1		0.00
Antenatal maternal smoking^a,b,d^	408	39.0		3,980	32.4		0.00
Antenatal maternal smoking, no. of cigarettes per day^a,b,d^							0.00
0	639	61.0		8,311	67.6		
1–9	106	10.1		1,041	8.5		
10–19	171	16.3		1,725	14.0		
≥20	131	12.5		1,214	9.9		
Socioeconomic status^b,e^							0.81
Management/professional	299	31.6		3,655	32.9		
Intermediate	178	18.8		2,148	19.3		
Self-employed	38	4.0		464	4.2		
Supervisory/technical	55	5.8		663	6.0		
Semiroutine/routine	375	39.7		4,179	37.6		
Breastfed^a,b,f^	688	65.6		8,493	69.0		0.02
White ethnicity^b,g^	881	84.1		10,416	84.8		0.53
Cesarean delivery^a,b,h^	458	43.7		2,516	20.5		0.00
Admitted to neonatal unit^a,b,i^	541	51.6		710	5.8		0.00
Length of stay after birth, days^a^			17.6 (24.3)			3.1 (5.8)	0.00
Exposure to smoking after birth	317	30.2		3,436	27.9		0.11
Atopy at any age	623	59.4		7,377	59.9		0.73
Asthma diagnosis^a^	339	32.3		2,969	24.1		0.00
Maternal age at child’s birth, years			29.1 (6.1)			28.8 (5.8)	0.16
Maternal history of atopy							0.13
Missing	1	0.0		9	0.0		
Asthma and eczema	73	7.0		697	5.7		
Asthma or eczema	243	23.2		2,716	22.1		
None	732	69.8		8,885	72.2		
Maternal prepregnancy BMI^j^			23.8 (5.2)			23.8 (4.4)	0.86
Maternal prepregnancy BMI category^a^							0.00
Refusal	0	0.0		2	0.0		
Not available	91	8.7		960	7.8		
Underweight	74	7.1		593	4.8		
Normal weight	613	58.4		7,396	60.1		
Overweight	165	15.7		2,368	19.2		
Obese	92	8.8		906	7.4		
Morbidly obese	14	1.3		82	0.7		
Damp or condensation exposure^b,k^	126	12.0		1,654	13.5		0.18
Pollution, grime, and environmental problems^b,l^							0.51
Very common	70	6.7		749	6.1		
Fairly common	150	14.5		1,943	15.9		
Not very common	403	38.3		4,773	39.2		
Not at all common	415	40.0		4,718	38.7		
Number of siblings in household^a^			0.86 (1.1)			0.93 (1.0)	0.03
Childcare^a,b,m^							0.00
Formal	135	12.9		1,758	14.3		
Informal	284	27.2		3,882	31.6		

Abbreviation: BMI, body mass index; IUGR, intrauterine growth restriction; SD, standard deviation.

^a^*P* < 0.05 between the term and preterm children with wheezing phenotype data.

^b^ Data was missing for some of the variables.

^c^ The total number of preterm children with data was 1,048. The total number of term children with data was 12,300.

^d^ The total number of preterm children with data was 1,047. The total number of term children with data was 12,291.

^e^ The total number of preterm children with data was 945. The total number of term children with data was 11,109.

^f^ The total number of term children with data was 12,306.

^g^ The total number of preterm children with data was 1,048. The total number of term children with data was 12,284.

^h^ The total number of preterm children with data was 1,048. The total number of term children with data was 12,252.

^i^ The total number of preterm children with data was 1,048.

^j^ Weight (kg)/height(m)^2^.

^k^ The total number of term children with data was 12,285.

^l^ The total number of preterm children with data was 1,038. The total number of term children with data was 12,183.

^m^ The total number of preterm children with data was 1,043. The total number of term children with data was 12,183.

A total of 794 (75.7%) and 255 (24.3%) of preterm-born children and 9,526 (77.4%) and 2,781 (22.6%) of term-born children had wheezing data at all 4 or at 3 time points, respectively. Recent wheeze was more common at all time points in the preterm-born group than in the term-born group, although the association was weaker at 11 years (Table 2). In general, there was a gradient, with odds ratios for recent wheeze increasing with decreasing gestation (Web Table 2).

**Table 2.** Unadjusted Association Between Preterm or Term Birth and Recent Wheezing (in the Previous 12 Months) at Each Evaluated Age Among Children Born During 2000–2002, Millennium Cohort Study, United Kingdom

Age, years	Preterm Born			Term Born			OR	95% CI	*P* Value
	No. With Recent Wheeze	Total No.	%	No. With Recent Wheeze	Total No.	%			
3^a^	259	984	26.3	2,228	11,584	19.2	1.5	1.3, 1.7	<0.001
5^a^	232	1,022	22.7	1,907	12,003	15.9	1.6	1.3, 1.8	<0.001
7^a^	150	990	15.2	1,402	11,743	11.9	1.3	1.1, 1.6	<0.005
11^a^	131	945	13.9	1,314	11,117	11.8	1.2	1.0, 1.5	0.06
Any time point	443	1,049	42.2	3,993	12,307	32.4	1.5	1.3, 1.7	<0.001

Abbreviations: CI, confidence interval; OR, odds ratio.

^a^ There is missing data for some of the variables.

**Figure 1.**
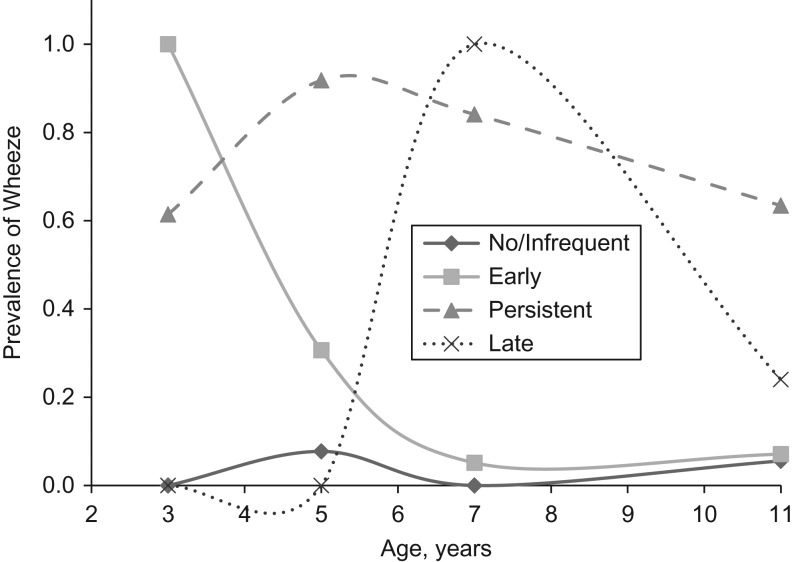
Wheezing phenotypes for preterm-born children born during 2000–2002, Millennium Cohort Study, United Kingdom.

Four phenotypes were defined, as shown in Figure 1 (see Web Appendix 1 for their derivation):

- No wheeze/infrequent wheeze: no or infrequent wheezing through the 4 time points. Wheezing at none of the time points or at 1 time point only.
- Early wheeze: wheezing reported at 3 years of age and disappearing by 7 or 11 years of age.
- Persistent wheeze: wheezing that persisted throughout the study period.
- Late wheeze: no wheeze reported before the age of 7 years but developing at age 7 years or beyond.

Table 3 compares the wheezing phenotypes between the preterm- and term-born children. Preterm-born children were more likely to develop early wheeze (odds ratio = 1.6, 95% confidence interval: 1.3, 1.9; *P* < 0.001) and persistent wheeze (odds ratio = 1.6, 95% confidence interval: 1.3, 1.9; *P* < 0.001) but not late wheeze (odds ratio = 1.0, 95% confidence interval: 0.7, 1.5; *P* = 0.90) when compared with term-born children.

**Table 3.** Unadjusted Associations Between Preterm or Term Birth and Wheezing Phenotypes Among Children Born During 2000–2002, Millennium Cohort Study, United Kingdom

Wheezing Phenotype	Preterm Born		Term Born		Total No.	OR	95% CI	*P* Value
	No.	%	No.	%				
No wheeze	693	66.1	9,193	74.7	9,886	1.0	Referent	
Early wheeze	189	18.0	1,597	13.0	1,786	1.6	1.3, 1.9	<0.001
Persistent wheeze	137	13.1	1,131	9.2	1,268	1.6	1.3, 1.9	<0.001
Late wheeze	30	2.9	386	3.1	416	1.0	0.7, 1.5	0.90
Total	1,049		12,307		13,356			

Abbreviations: CI, confidence interval; OR, odds ratio.

Although most demographic characteristics were similar between the different wheezing phenotypes (Web Table 3), birth weight, gestational age at birth, antenatal maternal smoking, neonatal unit admissions, length of hospital stay, asthma diagnosis, maternal atopy, and child’s atopy were different between the wheezing phenotypes, although differences for gestational age were marginal. Furthermore, antenatal smoking and neonatal unit admission were highest in the late-wheeze group; antenatal smoking was lowest in the persistent-wheeze group; and length of hospital stay was highest in the early-wheeze group. Atopy and asthma diagnosis were highest in the persistent-wheeze group.

Next, all early-life factors and characteristics were included in an initial multinomial model, and only those suggestive of an association were included in the final multinomial model for preterm (Table 4) and term groups (Table 5). Preterm- and term-born children who were exposed to antenatal maternal smoking, were male, or who had atopy had higher odds ratios for all the wheezing phenotypes. Formal childcare was associated with higher odds ratios for early wheeze in both preterm- and term-born children. The association between term-born children and antenatal maternal smoking was stronger, but the odds ratios were generally low. The strongest association for atopy for both preterm- and term-born children was with persistent wheeze. Preterm children born at 24–32 weeks’ gestation had higher odds ratios for all the wheezing phenotypes. The 24–32 weeks’ gestation band had higher odds ratios for all phenotypes compared with the 33–34 weeks’ gestation group. Preterm children born with IUGR had higher odds ratios for all the wheezing phenotypes. However, IUGR in term-born children led to a slightly increased risk of early and persistent wheeze but a lower risk of late wheeze. Maternal atopy was associated with higher odds ratio for persistent wheeze in the preterm group and higher odds ratios for all wheezing phenotypes in term-born children. Breastfeeding in preterm-born children was weakly associated with lower odds ratios for early and persistent but not late wheeze, which had a higher odds ratio; in term-born children, breastfeeding was weakly associated with lower odds ratios for all the wheezing phenotypes. The small numbers in the mother’s age bands and mother’s body mass index groups made it difficult to interpret the results. In term-born children, only cesarean delivery, exposure to damp, or having siblings were weakly associated with an increased risk of early and persistent wheeze.

**Table 4.** Adjusted Associations Between Early Risk Factors and Characteristics and Wheezing Phenotypes Among Preterm-Born Children (Complete Cases Only), Using the No/Infrequent-Wheezing Phenotype as the Reference Category, Among Children Born During 2000–2002, Millennium Cohort Study, United Kingdom

Early Risk Factor	Total No.^a^	Early Wheeze (*n* = 160)			Persistent Wheeze (*n* = 113)			Late Wheeze (*n* = 22)		
		OR	95% CI	*P* Value	OR	95% CI	*P* Value	OR	95% CI	*P* Value
Mother’s BMI category										
Underweight	53	2.124	1.053, 4.285	0.04	1.291	0.523, 3.182	0.58	1.882	0.390, 9.085	0.43
Normal	546	1.000	Referent		1.000	Referent		1.000	Referent	
Overweight	156	1.843	1.170, 2.901	0.01	1.135	0.633, 2.033	0.67	0.284	0.037, 2.212	0.23
Obese	84	1.006	0.505, 2.004	0.99	1.389	0.696, 2.775	0.35	1.842	0.567, 5.984	0.31
Morbidly obese	14	1.679	0.463, 6.085	0.43	0.960	0.186, 4.958	0.96	N/A		.
Gestation band, weeks										
24–32	158	2.010	1.257, 3.215	0.00	1.798	1.044, 3.098	0.03	2.458	0.897, 6.730	0.08
33–34	202	1.090	0.696, 1.708	0.71	0.822	0.476, 1.420	0.48	0.898	0.276, 2.926	0.86
35–36	493	1.000	Referent		1.000	Referent		1.000	Referent	
Breastfeeding										
Yes	571	0.676	0.457, 1.00	0.05	0.816	0.514, 1.298	0.39	1.288	0.471, 3.518	0.62
No	282	1.000	Referent		1.000	Referent		1.000	Referent	
IUGR										
Yes	117	1.619	0.981, 2.669	0.06	1.352	0.724, 2.528	0.34	1.248	0.345, 4.513	0.74
No	736	1.000	Referent		1.000	Referent		1.000	Referent	
Antenatal maternal smoking										
Yes	325	1.670	1.131, 2.466	0.01	1.482	0.938, 2.342	0.09	2.153	0.850, 5.453	0.11
No	528	1.000	Referent		1.000	Referent		1.000	Referent	
Childcare										
Formal	131	1.709	1.008, 2.897	0.05	1.094	0.572, 2.093	0.79	1.059	0.270, 4.152	0.93
Informal	260	1.091	0.715, 1.644	0.69	0.938	0.576, 1.529	0.80	0.674	0.233, 1.946	0.47
None	462	1.000	Referent		1.000	Referent		1.000	Referent	
Mother’s age band, years										
≤19	52	0.843	0.335, 2.122	0.72	1.737	0.708, 4.259	0.23	0.570	0.065, 4.970	0.61
20–24	113	1.793	0.996, 3.227	0.05	1.623	0.801, 3.290	0.18	0.645	0.129, 3.230	0.59
25–29	254	1.000	Referent		1.000	Referent		1.000	Referent	
30–34	277	1.138	0.697, 1.859	0.61	1.558	0.901, 2.694	0.11	1.120	0.394, 3.184	0.83
35–39	131	1.803	1.025, 3.171	0.04	1.481	0.735, 2.985	0.27	0.630	0.125, 3.171	0.58
≥40	26	2.308	0.873, 6.099	0.09	0.903	0.188, 4.329	0.90	1.754	0.192, 16.070	0.62
Maternal atopy										
Yes	254	0.984	0.650, 1.490	0.94	1.569	1.006, 2.447	0.05	0.976	0.363, 2.622	0.96
No	599	1.000	Referent		1.000	Referent		1.000	Referent	
Child’s sex										
Male	440	1.12	0.775, 1.618	0.55	1.803	1.169, 2.779	0.01	1.566	0.643, 3.814	0.32
Female	413	1.000	Referent		1.000	Referent		1.000	Referent	
Child atopy										
Yes	507	1.487	1.019, 2.169	0.04	4.432	2.597, 7.563	0.00	2.416	0.906, 6.440	0.08
No	346	1.000	Referent		1.000	Referent		1.000	Referent	

Abbreviation: BMI, body mass index; CI, confidence interval; IUGR, intrauterine growth restriction; OR, odds ratio.

^a^ Total number includes those in no/infrequent-wheeze group.

**Table 5.** Adjusted Associations Between Early Risk Factors and Characteristics and Wheezing Phenotypes Among Term-Born Children (Complete Cases Only), Using the No/Infrequent-Wheezing Phenotype as the Reference Category, Among Children Born During 2000–2002, Millennium Cohort Study, United Kingdom

Early Risk Factor	Total No.^a^	Early Wheeze (*n* = 1,325)			Persistent Wheeze (*n* = 943)			Late Wheeze (*n* = 309)		
		OR	95% CI	*P* Value	OR	95% CI	*P* Value	OR	95% CI	*P* Value
Mother’s BMI category										
Underweight	474	1.294	1.001, 1.673	0.05	0.906	0.636, 1.291	0.59	0.872	0.466, 1.630	0.67
Normal	6,676	1.000	Referent		1.000	Referent		1.000	Referent	
Overweight	2,122	1.086	0.934, 1.263	0.28	1.300	1.099, 1.538	0.00	1.163	0.877, 1.543	0.29
Obese	839	1.229	0.995, 1.519	0.06	1.214	0.948, 1.556	0.12	1.280	0.857, 1.911	0.23
Morbidly obese	68	1.761	0.950, 3.266	0.07	1.096	0.480, 2.501	0.83	1.471	0.446, 4.854	0.53
Pollution										
Very common	607	0.981	0.756, 1.272	0.88	1.213	0.909, 1.619	0.19	0.920	0.561, 1.510	0.74
Fairly common	1,520	1.052	0.881, 1.256	0.57	1.219	0.995, 1.493	0.06	0.814	0.571, 1.162	0.26
Not very common	3,932	0.992	0.868, 1.134	0.91	1.045	0.890, 1.226	0.59	0.792	0.612, 1.026	0.08
Not at all common	4,120	1.000	Referent		1.000	Referent		1.000	Referent	
Damp										
Yes	1,270	1.100	0.925, 1.310	0.28	1.212	0.992, 1.480	0.06	0.911	0.629, 1.320	0.62
No	8,909	1.000	Referent		1.000	Referent		1.000	Referent	
Childcare										
Formal	1,626	1.253	1.038, 1.512	0.02	0.742	0.585, 0.940	0.01	0.918	0.639, 1.320	0.65
Informal	3,573	1.052	0.918, 1.205	0.47	0.912	0.779, 1.067	0.25	0.924	0.711, 1.201	0.56
None	4,980	1.000	Referent		1.000	Referent		1.000	Referent	
Breastfeeding										
Yes	7,142	0.849	0.743, 0.971	0.02	0.825	0.704, 0.967	0.02	0.855	0.657, 1.112	0.24
No	3,037	1.000	Referent		1.000	Referent		1.000	Referent	
IUGR										
Yes	934	1.198	0.986, 1.455	0.07	1.092	0.859, 1.387	0.47	0.366	0.199, 0.675	0.00
No	9,245	1.000	Referent		1.000	Referent		1.000	Referent	
Antenatal maternal smoking										
Yes	3,316	1.207	1.058, 1.378	0.01	1.206	1.031, 1.410	0.02	1.427	1.105, 1.842	0.01
No	6,863	1.000	Referent		1.000	Referent		1.000	Referent	
Mother’s age band, years										
≤19	563	1.269	0.968, 1.664	0.09	0.726	0.502, 1.050	0.09	0.675	0.365, 1.247	0.21
20–24	1,610	1.212	1.010, 1.454	0.04	1.170	0.945, 1.447	0.15	0.687	0.455, 1.037	0.07
25–29	2,854	1.000	Referent		1.000	Referent		1.000	Referent	
30–34	3,315	0.888	0.758, 1.041	0.14	0.842	0.699, 1.015	0.07	1.180	0.876, 1.588	0.28
35–39	1,596	0.794	0.648, 0.974	0.03	0.964	0.768, 1.211	0.75	1.288	0.897, 1.851	0.17
40 plus	241	1.381	0.949, 2.008	0.09	1.470	0.959, 2.255	0.08	1.863	0.936, 3.707	0.08
Social class category										
1 (highest)	3,408	0.833	0.702, 0.990	0.04	1.007	0.825, 1.230	0.94	0.815	0.592, 1.122	0.21
2	1,991	0.869	0.730, 1.033	0.11	0.989	0.807, 1.213	0.92	0.855	0.613, 1.192	0.35
3	420	0.898	0.653, 1.234	0.51	1.045	0.722, 1.512	0.82	0.314	0.126, 0.784	0.01
4	615	0.763	0.584, 0.997	0.05	1.050	0.784, 1.405	0.75	0.774	0.459, 1.306	0.34
5 (lowest)	3,745	1.000	Referent		1.000	Referent		1.000	Referent	
Maternal atopy										
Yes	2,903	1.537	1.355, 1.744	0.00	1.604	1.386, 1.856	0.00	1.228	0.955, 1.578	0.11
No	7,276	1.000	Referent		1.000	Referent		1.000	Referent	
Siblings										
Yes	5,865	1.251	1.092, 1.433	0.00	1.142	0.975, 1.338	0.10	0.948	0.730, 1.229	0.69
No	4,314	1.000	Referent		1.000	Referent		1.000	Referent	
Cesarean delivery										
Yes	2,099	1.069	0.920, 1.241	0.38	1.204	1.017, 1.425	0.03	0.865	0.644, 1.161	0.33
No	8,080	1.000	Referent		1.000	Referent		1.000	Referent	
Sex										
Male	5,138	1.266	1.125, 1.426	0.00	1.640	1.423, 1.890	0.00	1.278	1.016, 1.609	0.04
Female	5,041	1.000	Referent		1.000	Referent		1.000	Referent	
Child atopy										
Yes	6,122	1.477	1.304, 1.672	0.00	4.559	3.773, 5.509	0.00	2.665	2.031, 3.496	0.00
No	4,057	1.000	Referent		1.000	Referent		1.000	Referent	

Abbreviation: BMI, body mass index; CI, confidence interval; IUGR, intrauterine growth restriction; OR, odds ratio.

^a^ Total number includes those in no/infrequent wheeze group.

## DISCUSSION

Using a well-established cohort with longitudinal data, we noted that rates of recent wheeze in preterm-born children, when compared with term-born children, were higher at each time point (although the association was weaker at 11 years), as previously reported (8, 32). We also defined 4 wheezing phenotypes as recently reported in largely term-born children in the MCS (33). The odds ratios for early and persistent wheeze were greater in the preterm group, but late wheeze was similar in both groups. Early-life factors and characteristics, especially antenatal maternal smoking, atopy, and male sex, were associated with wheezing phenotypes in both preterm and term groups, and breastfeeding was associated with decreased rates of wheezing phenotypes in both groups, except for late wheeze in the preterm-born children, although the magnitude of association according to various early-life factors and characteristics varied between the groups. However, prematurity was associated with higher rates of wheezing in the early and persistent groups but not the late-wheeze group, suggesting that delivery at an early stage of lung development is a risk factor for the development of certain wheezing phenotypes.

In line with previous reports (8), we also noted a higher rate of recent wheeze among preterm-born children than among term-born children, although there was similar development of wheezing phenotypes in both groups. It has been assumed that respiratory symptoms and lung function deficits commence in infancy and continue into later life, given the accepted concept of tracking of lung function (34). This concept is perhaps not surprising given the delivery of preterm infants at an early stage of lung development (19). It has been assumed that postnatal lung growth and development, especially with exposure to noxious substances such as supplemental oxygen therapy (35), is abnormal, placing these children at future risk of chronic obstructive pulmonary disease (18). We noted that the majority of preterm-born children commenced their respiratory symptoms in early childhood, but there was a group who had late wheeze comparable to term-born children. The group of very preterm-born children had a higher risk of all the wheezing phenotypes. Encouragingly, early wheeze was ameliorated among more than half of the children. Taken together, these data suggest that wheezing in preterm-born children is a heterogeneous disease process in which continuing growth and remodeling of the airways and parenchyma in childhood results in decreased symptoms in many (36). Because some wheezing phenotypes in largely term-born children are associated with lung function deficits (especially persistent wheeze) (12, 14, 16, 17), it will be important to investigate whether similar lung deficits are associated with particular wheezing phenotypes among preterm-born children. As suggested for asthma, the underlying mechanisms or endotypes (37, 38) and responses to treatment might be different for each wheezing phenotype in preterm-born children, although treatment for prematurity-associated wheeze remains uncertain (39).

We had anticipated that wheezing would be persistent in very preterm-born children, especially given that many of these infants are exposed to noxious substances (35). However, we were surprised to note similar gestational age between the different phenotypes. Because we did not have comprehensive early neonatal data, we were unable to determine whether early-life factors and exposures could be associated with the development of a particular wheeze phenotype. Nevertheless, the combination of these factors often results in development of bronchopulmonary dysplasia in infancy/chronic lung disease of prematurity, which is associated with greater respiratory symptoms and lung function decrements (2).

When only the early-life factors and characteristics were investigated, similar associations were observed between antenatal maternal smoking, formal childcare, male sex, and child’s atopy and the wheezing phenotypes in both groups. Differences, albeit marginal, were observed for IUGR, breastfeeding, cesarean delivery, having siblings, exposure to damp, and maternal atopy and the wheezing phenotypes for preterm- and term-born children. All wheezing phenotypes in both groups were associated with atopy, but the association was greatest for persistent wheeze in both groups. Similar observations have been made for phenotypes in a birth cohort of mainly term-born children (14), and a systematic review reporting factors predicting persistence of early wheeze noted that atopy was among the most frequently identified factors (40).

Wheezing phenotypes have been described in cohorts of mainly term-born children (12–15). Our observations suggest that prematurity is also associated with differing phenotypes, with some that resolve or decrease in prevalence (early), continue (persistent), or develop later (late). Caudri et al. (41) reported additional risk factors associated with the wheezing phenotype; interestingly, cesarean delivery was not associated. This is in line with our recent study reporting that cesarean delivery was not strongly associated, but the relative immaturity of the early-term born infant was important (10). However, we did observe an association between cesarean delivery and early and persistent wheeze in term-born children.

Modeling for the preterm-born children showed only that child’s atopy and antenatal maternal smoking were associated with an increased risk of all the wheezing phenotypes. This is in line with an analysis of 8 birth cohorts containing term- and preterm-born children, which concluded that there was an increased risk of wheeze and asthma among children who were exposed to maternal smoking during pregnancy but were not exposed after birth (31). In addition to the associations of in utero smoke with the child’s lung development, maternal smoking leads to a higher rate of preterm-birth (42). A study of mainly term-born children suggested that inherited factors are a primary cause of late-onset persistent wheeze. However, environmental exposure in early life might combine with inherited factors, resulting in early-onset persistent wheeze (30). Another study identified some modifiable factors in infancy, such as household dampness and breastfeeding (29).

The strength of the present study is the inclusion of a large number of preterm-born children with longitudinal data. We did not, however, have comprehensive early neonatal data, formal allergen testing, or lung function measures that would have enabled association of lung function deficits with particular wheezing phenotypes as observed in term-born children (43). A limitation of the present study is that early neonatal data were not available for the preterm group, including the need for mechanical ventilation, surfactant treatment, development of bronchopulmonary dysplasia in infancy, and length of oxygen supplementation, especially given that these factors could potentially have been relevant to the types of wheezing phenotype that the preterm-born child might have developed. A limitation of this prospective study is that the wheeze data is self-reported. However, the questions used are taken from the International Study of Asthma and Allergies in Childhood (44). The questionnaire is a widely used, well-respected, and validated questionnaire. Jenkins et al. (45) reported that the questionnaire showed high agreement with doctor-diagnosed asthma symptoms. Shaw et al. (46) also validated the questionnaire and reported that it was effective in measuring the prevalence of bronchial hyperresponsiveness. Investigators in Brazil concluded that the asthma section was reproducible and could separate out asthmatic individuals and controls (47). In agreement, Finnish investigators reported that the International Study of Asthma and Allergies in Childhood questionnaire was highly validated (48). Loss to follow-up was a further limitation.

In conclusion, to our knowledge, we have defined wheezing phenotypes for preterm-born children for the first time. Encouragingly, a large proportion of preterm-born children have early wheeze that improves with age, although this might not be true for very preterm-born children, as reported in some other studies. Early-life factors and characteristics appeared to have similar associations with wheezing phenotypes in both preterm and term groups. However, the odds ratios were greater in the preterm group, especially for early and persistent wheeze. The underlying mechanisms of why preterm-born children develop lung disease are unclear, but for both groups it is clear that the avoidance of risk factors, such as antenatal maternal smoking, or the exposure to beneficial factors, such as breastfeeding, is important.

## Supplementary Material

Web Material

## Abbreviations

IUGR: intrauterine growth restriction
MCS: Millennium Cohort Study
