# Supplementary material for: Comparison of the Associations of Early-Life Factors on Wheezing Phenotypes in Preterm-Born Children and Term-Born Children
Source: Am J Epidemiol. 2019 Jan 22;188(3):527–36. doi: 10.1093/aje/kwy268 (PMC6395162; doi:10.1093/aje/kwy268)

Web Material

Comparison of the Associations of Early-Life Factors on Wheezing Phenotypes in Preterm-Born Children and Term-Born Children

Sarah J Kotecha, W John Watkins, John Lowe, Raquel Granell, A John Henderson, and Sailesh Kotecha.

Table of contents

**Web Appendix 1**	**Page 2**
**Web Appendix 2**	**Page 3**
**Web Table 1**	**Page 4**
**Web Table 2**	**Page 6**
**Web Table 3**	**Page 7**
**Web Figure 1**	**Page 10**

Web Appendix 1

METHODS

MCS is a cohort of 19,517 children born in the UK between 2000-2002. The children were selected through the Child Benefit system with over-sampling from Wales, Scotland and Northern Ireland. Oversampling also occurred from areas with higher concentrations of Black and Asian families, and from deprived areas.

All data were collected at face-to-face interviews. At nine months of age, data were collected on pregnancy, birth and early life including breast feeding (breast milk intake for any period of time), any antenatal maternal smoking and socio-economic status (based on main carer's last known job), delivery by caesarean section (CS), exposure to damp or condensation, exposure to pollution, grime and environmental problems, number of siblings, childcare use (informal e.g. family members and formal e.g. childminder, nursery etc.), maternal history of atopy (none, asthma and eczema, asthma or eczema), maternal pre-pregnancy body mass index (BMI) (underweight, normal, overweight, obese, morbidly obese), maternal age at birth of child. At 3, 5, 7 and 11 years of age, longitudinal data were collected for respiratory symptoms (including wheeze ever and recent wheeze – defined as parental reporting of wheezing or whistling in the chest – in the last 12 months), asthma-diagnosis (based on parental reports) and child's atopic diseases (diagnosis of eczema and hay fever), exposure to smoking (positive if exposure occurred at any time-point in the same room as the child). Ethnicity was coded as Caucasian or non-Caucasian.

Statistical Analysis

We, used LatentGOLD 5.1 (Statistical Innovations, Boston, Ma., USA) to define the optimal number of phenotypes to study. Children reporting recent-wheeze for at least three time-points were assigned a 4-digit string with the digits assigned 1 or 0 for recent-wheeze at 3, 5, 7 and 11 years. Thus "0000" signified never wheezed and "1111" signified wheezing at all-time points. Where there were three reports of recent-wheeze, the missing time period was assigned an "asterisk". For the term and preterm-born children who had data available at 3 or 4 time points 4 classes were chosen as the first non-significant p-value and the lowest BIC. The class posterior probability was used to assign each wheezing pattern to the class of wheezing-phenotype which they had the highest probability of belonging to. The machine driven approach was used for all the wheezing patterns.

Web Appendix 2

RESULTS

Web Table 1

Web Table 1 compares children who were or were not included. A greater percentage of preterm- and term-born children for whom wheezing data were not available were male, from lower socio-economic classes, had lower rates of breast-fed or asthma-diagnosis, less exposure to post-natal smoking, had younger mothers, a greater percentage of maternal BMIs were outside the normal weight range, were more likely to be from an ethnic minority, were more likely to be exposed to pollution, grime and environmental problems, had lower rates of being in formal or informal childcare but were less likely to be atopic. The term-born children not included also had lower birth-weight z-score and higher exposure to antenatal maternal smoking and had more siblings in the household.

Web Tables

Web Table 1 – Demographics of the Preterm- and Term-Born Children With and Without Wheezing-Phenotype Data. United Kingdom, Year of Birth 2000-2002

	Term children with wheezing phenotype data	Term children without wheezing phenotype data	Preterm children with wheezing phenotype data	Preterm children without wheezing phenotype data
**Number**	12,307	4,756	1,049	453
**Mean birth-weight (kg) (SD)$**	3.43 (0.51)	3.39 (0.51)	2.33 (0.68)	2.35 (0.72)
**Mean birth-weight (z-score) (SD)$**	-0.03 (1.00)	-0.08 (1.01)	0.01 (1.20)	0.04 (1.23)
**Mean gestation (weeks) (SD)$**	39.81 (1.27)	39.72 (1.29)	34.34 (2.43)	34.18 (2.68)
**IUGR at birth N (%)**	1,238/12,300 (10.1)	517/4,750 (10.9)	145/1,048 (13.8)	58/453 (12.8)
**Male N (%) $***	6,202/12,307 (50.4)	2,501/4,756 (52.6)	536/1,049 (51.1)	268/453 (59.2)
**Antenatal maternal smoking N (%)$**	3,980/12,291(32.4)	1,892/4,748 (39.8)	408/1,047 (39.0)	181/452 (40.0)
**Antenatal smoke exposure N (%)$**				
**none**	8,311/12,291 (67.6)	2,856/4,748 (60.2)	639/1,047 (61.0)	271/452 (60.0)
**1-9 cigarettes**	1,041/12,291 (8.5)	525/4,748 (11.1)	106/1,047 (10.1)	46/452 (10.2)
**10-19 cigarettes**	1,725/12,291 (14.0)	816/4,748 (17.2)	171/1,047 (16.3)	69/452 (15.3)
**>/= 20 cigarettes**	1,214/12,291 (9.9)	551/4,748 (11.6)	131/1,047 (12.5)	66/452 (14.6)
**Socio-economic status N (%)$***				
**Management/Professional**	3,655/11,109 (32.9)	841/3,997 (21.0)	299/945 (31.6)	89/389 (22.9)
**Intermediate**	2,148/11,109 (19.3)	733/3,997 (18.3)	178/945 (18.8)	69/389 (17.7)
**Self employed**	464/11,109 (4.2)	142/3,997 (3.6)	38/945 (4.0)	7/389 (1.8)
**Supervisory/Technical**	663/11,109 (6.0)	252/3,997 (6.3)	55/945 (5.8)	25/389 (6.4)
**Semi routine/routine**	4,179/11,109 (37.6)	2,029/3,997 (50.8)	375/945 (39.7)	199/389 (51.2)
**Breast fed N (%) $***	8,493/12,306 (69.0)	2,740/4,753 (57.6)	688/1,049 (65.6)	267/453 (58.9)
**Ethnicity white N (%) $***	10,416/12,284 (84.8)	3,724/4,751 (78.4)	881/1,048 (84.1)	353/453 (77.9)
**Caesarean section N (%)**	2,516/12,252 (20.5)	1,000/4,739 (21.1)	458/1,048 (43.7)	194/453 (42.8)
**Admitted to NNU N (%)$**	710/12,307 (5.8)	297/4,751 (6.3)	541/1,048 (51.6)	223/453 (49.2)
**Length of stay after birth (days) (SD)***	3.12 (5.81)	3.25 (6.09)	17.63 (24.31)	21.45 (34.91)
**Exposure to smoking after birth N (%)$***	3,436/12,307 (27.9)	1,090/4,755 (22.9)	317/1,049 (30.2)	103/452 (22.8)
**Atopy at any age N (%)$***	7,377/12,307 (59.9)	1,253/2,978 (42.1)	623/1,049 (59.4)	116/291 (39.9)
**Asthma-diagnosis N (%)$***	2,969/12,307 (24.1)	527/2,973 (17.7)	339/1,049 (32.3)	69/291 (23.7)
**Maternal age at child's birth (yrs) (SD) *$**	28.8 (5.8)	27.2 (6.1)	29.1 (6.1)	27.3 (6.4)
**Maternal history of atopy N (%)**				
**Missing**	9/12,307 (0.0)	1/4,756 (0.0)	1/1,049 (0.0)	0/453 (0)
**Asthma and Eczema**	697/12,307 (5.7)	264/4,756 (5.6)	73/1,049 (7.0)	28/453 (6.2)
**Asthma or Eczema**	2,716/12,307 (22.1)	980/4,756 (20.6)	243/1,049 (23.2)	83/453 (18.3)
**None**	8,885/12,307 (72.2)	3,511/4,756 (73.8)	732/1,049 (69.8)	342/453 (75.5)

**Maternal's BMI before child's pregnancy (kg/m²) (SD)** *$	23.8 (4.4)	23.4 (4.3)	23.7 (5.2)	23.1 (4.9)
**Maternal BMI group before pregnancy N (%)***$				
**Refusal**	2/12,307 (0)	2/4,756 (0)	0/1,049 (0)	0/453 (0)
**Not applicable**	960/12,307 (7.8)	480/4,756 (10.1)	91/1,049 (8.7)	51/453 (11.3)
**Underweight**	593/12,307 (4.8)	307/4,756 (6.5)	74/1,049 (7.1)	44/453 (9.7)
**Normal weight**	7,396/12,307 (60.1)	2,798/4,756 (58.8)	613/1,049 (58.4)	252/453 (55.6)
**Overweight**	2,368/12,307 (19.2)	809/4,756 (17.0)	165/1,049 (15.7)	78/453 (17.2)
**Obese**	906/12,307 (7.4)	338/4,756 (7.1)	92/1,049 (8.8)	22/543 (4.9)
**Morbidly Obese**	82/12,307 (0.7)	22/4,756 (0.5)	14/1,049 (1.3)	6/453 (1.3)
**Damp or condensation exposure N (%)**	1,654/12,285 (13.5)	682/4,738 (14.4)	126/1,049 (12.0)	69/452 (15.3)
**Pollution, grime and environmental problems N (%)***$				
**Very common**	749/12,183 (6.1)	320/4,685 (6.8)	70/1,038 (6.7)	39/449 (8.7)
**Fairly common**	1,943/12,183 (15.9)	818/4,685 (17.5)	150/1,038 (14.5)	72/449 (16.0)
**Not very common**	4,773/12,183 (39.2)	1,789/4,685 (38.2)	403/1,038 (38.3)	195/449 (43.4)
**Not at all common**	4,718/12,183 (38.7)	1,758/4,685 (37.5)	415/1,038 (40.0)	143/449 (31.8)
**Number of siblings in household N**$	0.93 (1.0)	0.97 (1.1)	0.86 (1.1)	0.87 (1.1)
**Childcare N (%)***$				
**Formal**	1,758/12,276 (14.3)	376/4,749 (8.0)	135/1,043 (12.9)	35/451 (7.8)
**Informal**	3,882/12,276 (31.6)	1,248/4,749 (26.3)	284/1,043 (27.2)	105/451 (23.3)

IUGR= intrauterine growth restriction

$ ***P*** <0.05 between those with and without wheezing-phenotype data for the term children

* ***P*** <0.05 between those with and without wheezing-phenotype data for the preterm children

Web Table 2 – Unadjusted Odds Ratio of Recent Wheeze at Each Age for Each Gestation Band Against Term. United Kingdom, Year of Birth 2000-2002.

Gestation group	3 years of age (N)	Odds ratio (95% CI)	*P* -value
**24-32 weeks**	62/181 (34.3%)	2.2 (1.6, 3.0)	<0.0001
**33-34 weeks**	62/233 (26.6%)	1.5 (1.1, 2.0)	0.0051
**35-36 weeks**	135/570 (23.7%)	1.3 (1.1, 1.6)	0.0089
**Term**	2,228/11,584 (19.2%)	ref	Ref
**Gestation group**	**5 years of age (N)**	**Odds ratio (95% CI)**	***P* -value**
**24-32 weeks**	47/186 (25.3%)	1.8 (1.3, 2.5)	0.0006
**33-34 weeks**	58/240 (24.2%)	1.7 (1.3, 2.3)	0.0006
**35-36 weeks**	127/596 (21.3%)	1.4 (1.2, 1.8)	0.0005
**Term**	1,907/12,003 (15.9%)	ref	ref
**Gestation group**	**7 years of age (N)**	**Odds ratio (95% CI)**	***P* -value**
**24-32 weeks**	36/178 (20.2%)	1.9 (1.3, 2.7)	0.0009
**33-34 weeks**	31/232 (13.4%)	1.1 (0.8, 1.7)	0.51
**35-36 weeks**	83/580 (14.3%)	1.2 (1.0, 1.6)	0.09
**Term**	1,402/11,743 (11.9%)	ref	Ref
**Gestation group**	**11 years of age (N)**	**Odds ratio (95% CI)**	***P* -value**
**24-32 weeks**	33/177 (18.6%)	1.7 (1.2,2.5)	0.006
**33-34 weeks**	31/221 (14.0%)	1.2 (0.8,1.8)	0.32
**35-36 weeks**	67/547 (12.2%)	1.0 (0.8, 1.4)	0.76
**Term**	1,314/11,117 (11.8%)	ref	Ref
**Gestation group**	**Any wheeze at any time point (N)**	**Odds ratio (95% CI)**	**p-value**
**24-32 weeks**	101/192 (52.6%)	2.3 (1.7, 3.1)	<0.0001
**33-34 weeks**	102/249 (41.0%)	1.4 (1.2, 1.9)	0.0047
**35-36 weeks**	240/608 (39.5%)	1.4 (1.1, 1.6)	0.0003
**Term**	3,993/12,307 (32.4%)	ref	ref

Web Table 3 – Characteristics for the Wheezing-Phenotypes for the Preterm Children Only. United Kingdom, Year of Birth 2000-2002

	No-Wheeze n = 693	Early-Wheeze n = 189	Persistent-Wheeze n = 137	Late-Wheeze n = 30	All children with positive wheezing phenotype n = 356	*P* value between the four wheezing phenotype groups
**Mean birth-weight (kg) (SD)***	2.39 (0.65)	2.18 (0.76)	2.28 (0.69)	2.21 (0.59)	2.22(0.72)	0.00
**Mean birth-weight (z-score) (SD)**	0.07 (1.20)	-0.17 (1.26)	-0.05 (1.17)	0.05 (1.02)	-0.11 (1.21)	0.09
**Mean gestation (weeks) (SD)***	34.52 (2.21)	33.89 (2.90)	34.12 (2.59)	33.84 (2.66)	33.98 (2.76)	0.01
**IUGR at birth N (%)**	84/692 (12.1)	35/189 (18.5)	23/137 (16.8)	3/30 (10.0)	61/356 (17.1)	0.09
**Male N (%)**	343/693 (49.5)	95/189 (50.3)	82/137 (59.9)	16/30 (53.3)	193/356 (54.2)	0.17
**Antenatal maternal smoking N (%) ***	248/691 (35.9)	86/189 (45.5)	60/137 (43.8)	14/30 (46.7)	160/356 (44.9)	0.04
**Antenatal smoke exposure N(%)***						0.03
**none**	443/691 (64.1)	103/189 (54.5)	77/137 (56.2)	16/30 (53.3)	196/356 (55.1)	
**1-9 cigarette**	74/691 (10.7)	16/189 (8.5)	15/137 (10.9)	1/30 (3.3)	32/356 (9.0)	
**10-19 cigarette**	95/691 (13.7)	44/189 (23.3)	26/137 (19.0)	6/30 (20.0)	76/356 (21.3)	
**>/= 20 cigarette**	79/691 (11.4)	26/189 (13.8)	19/137 (13.9)	7/30 (23.3)	52/356 (14.6)	
**Socio-economic status N (%)**						0.50
**Management/Professional**	193/624 (30.9)	54/171 (31.6)	45/123 (36.6)	7/27 (25.9)	106/321 (33.0)	
**Intermediate**	131/624 (21.0)	28/171 (16.4)	13/123 (10.6)	6/27 (22.2)	47/321 (14.6)	
**Self employed**	25/624 (4.0)	8/171 (4.7)	3/123 (2.4)	2/27 (7.4)	13/321 (4.0)	
**Supervisory/Technical**	34/624 (5.4)	12/171 (7.0)	8/123 (6.5)	1/27 (3.7)	21/321 (6.5)	
**Semi routine/routine**	241/624 (38.6)	69/171 (40.4)	54/123 (43.9)	11/27 (40.7)	134/321 (41.7)	
**Breast-fed N (%)**	465/693 (67.1)	112/189 (59.3)	89/137 (65.0)	22/30 (73.3)	223/356 (62.6)	0.18
**Ethnicity white N (%)**	574/692 (82.9)	162/189 (85.7)	119/137 (86.9)	26/30 (86.7)	307/356 (86.2)	0.58
**Caesarean section N (%)**	290/693 (41.8)	93/188 (49.5)	62/137 (45.3)	13/30 (43.3)	168/355 (47.3)	0.30
**Admitted to NNU N (%)***	337/693 (48.6)	107/189 (56.6)	80/137 (58.4)	17/29 (58.6)	204/355 (57.5)	0.00
**Length of stay after birth (days) (SD) ***	15.54 (21.41)	22.50 (29.61)	20.83 (28.35)	20.60 (25.34)	21.70 (28.73)	0.00

**Exposure to smoking after birth N (%)**	197/693 (28.4)	60/189 (31.7)	48/137 (35.0)	12/30 (40.0)	120/356 (33.7)	0.25
**Asthma-diagnosis N (%)***	111/693 (16.0)	91/189 (48.1)	124/137 (90.5	13/30 (43.3)	228/356 (64.0)	0.00
**Atopy at any age N (%)***	377/693 (54.4)	110/189 (58.2)	112/137 (81.8)	24/30 (80.0)	246/356 (69.1)	0.00
**Maternal age at child's birth (yrs) (SD)**	28.93 (5.93)	29.75 (6.60)	28.77 (6.43)	29.27 (6.88)	29.33 (6.56)	0.39
**Maternal history of atopy N (%)***						0.01
**Missing**	1/693 (0.0)	0/189 (0.0)	0/137 (0.0)	0/30 (0.0)	0/356 (0.0)	
**Asthma and Eczema**	38/693 (5.5)	12/189 (6.3)	19/137 (13.9)	4/30 (13.3)	35/356 (9.8)	
**Asthma or Eczema**	157/693 (22.7)	43/189 (22.8)	38/137 (27.7)	5/30 (16.7)	86/356 (24.2)	
**None**	497/693 (71.7)	134/189 (70.9)	80/137 (58.4)	21/30 (70.0	235/356 (66.0)	
**Maternal's BMI before pregnancy (kg/m²) (SD)**	23.56 (4.87)	24.25 (6.07)	23.98 (5.68)	23.16 (5.08)	24.06 (5.84)	0.39
**Maternal BMI group before pregnancy N (%)**						0.17
**Not applicable**	66/693 (9.5)	11/189 (5.8)	9/137 (6.6)	5/30 (16.7)	25/356 (7.0)	
**Underweight**	45/693 (6.5)	18/189 (9.5)	9/137 (6.6)	2/30 (6.7)	29/356 (8.1)	
**Normal weight**	414/693 (59.7)	99/189 (52.4)	82/137 (59.9)	18/30 (60.0)	199/356 (55.9)	
**Overweight**	101/693 (14.6)	42/189 (22.2)	21/137 (15.3)	1/30 (3.3)	64/356 (18.0)	
**Obese**	59/693 (8.5)	15/189 (7.9)	14/137 (10.2)	4/30 (13.3)	33/356 (9.3)	
**Morbidly Obese**	8/693 (1.2)	4/189 (2.1)	2/137 (1.5)	0/30 (0.0)	6/355 (1.7)	
**Damp or condensation exposure N(%)**	80/693 (11.5)	21/189 (11.1)	21/137 (15.3)	4/30 (13.3)	46/356 (12.9)	0.62
**Pollution, grime and environmental problems N (%)**						0.45
**Very common**	47/685 (6.9)	9/188 (4.8)	10/136 (7.4)	4/29 (13.8)	23/353 (6.5)	
**Fairly common**	91/685 (13.3)	36/188 (19.1)	19/136 (14.0)	4/29 (13.8)	59/353 (16.7)	
**Not very common**	278/685 (40.6)	66/188 (35.1)	49/136 (36.0)	10/39 (34.5)	125/353 (35.4)	
**Not at all common**	269/685 (39.3)	77/188 (41.0)	58/136 (42.6)	11/39 (37.9)	146/353 (41.4)	
**Number of siblings in household mean mean (SD)**	0.83 (1.03)	0.89 (1.18)	0.93 (1.10)	0.93 (1.02)	0.91 (1.13)	0.69

**Childcare N(%)**						0.81
**Formal**	85/690 (12.3)	30/187 (16.0)	16/136 (11.8)	4/30 (13.3)	50/353 (14.2)	
**Informal**	190/690 (27.5)	52/187 (27.8)	35/136 (25.7)	7/30 (23.3)	94/353 (26.6)	

IUGR=intrauterine growth restriction

*** *P* <0.05 between the four wheezing-phenotype groups**

There is missing data for some of the demographics

Web Figure 1 - Assumed Causal Pathway Linking Early Life Factors and Characteristics to Preterm Birth and/or Later Wheezing. United Kingdom, Year of Birth 2000-2002.

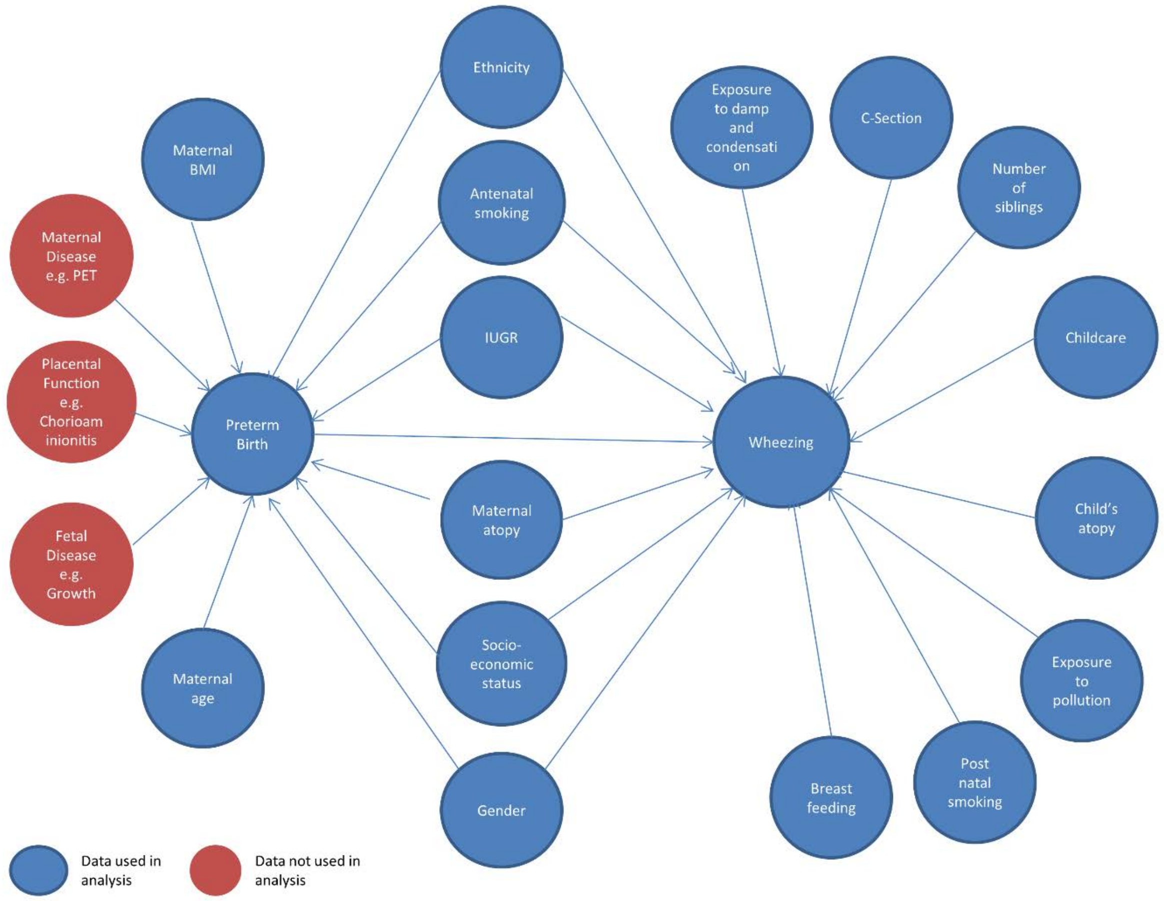

Supplement: Web Material [file kwy268_kotecha_web_material_final.pdf]
